# Alembic: a framework for converting disparate biological data into structured resources

**DOI:** 10.18699/vjgb-26-33

**Published:** 2026-04

**Authors:** I.V. Bezdvornykh, K.I. Yuditskiy, N.A. Cherkasov, A.A. Samsonova, A.A. Kanapin

**Affiliations:** Institute for Translational Biomedicine, Saint Petersburg State University, St. Petersburg, Russia; Institute for Translational Biomedicine, Saint Petersburg State University, St. Petersburg, Russia; Institute for Translational Biomedicine, Saint Petersburg State University, St. Petersburg, Russia; Institute for Translational Biomedicine, Saint Petersburg State University, St. Petersburg, Russia; Institute for Translational Biomedicine, Saint Petersburg State University, St. Petersburg, Russia

**Keywords:** natural language processing, biomedical text mining, semantic annotation, data harmonization, omics data integration, обработка естественных языков, анализ биомедицинскиx текстов, семантическая аннотация, гармонизация данных, интеграция омиксных даных

## Abstract

The imperative to re-analyze existing public sequencing data is central to modern biology, driven by new hypotheses and advanced analytical methods. However, this effort is critically hampered by the profound heterogeneity of repository data, particularly the non-standardized, free-text descriptions of biological experiments. This lack of structural and semantic homogeneity prevents systematic search, integration, and comparative analysis, effectively locking away the full potential of accumulated datasets. Advances in Natural Language Processing (NLP) offer a pivotal pathway to overcome this bottleneck by transforming unstructured text into computable, homogeneous information. The integrated Entrez database system, maintained by the National Center for Biotechnology Information (NCBI), provides sophisticated programmatic access via an API to primary sequencing data and its associated metadata, including detailed experimental descriptions. This interface enables researchers to identify and retrieve relevant data through keyword searches, including those based on gene names, and to apply modern NLP techniques to transform textual metadata into structured information. The output is formatted data ready for integration into local databases, accompanied by a systematic list of links for downloading primary files. The Alembic software package offers a comprehensive and automated solution for the entire workflow. Designed as a locally deployable client-server system, Alembic incorporates state-of-the-art transformer-based AI algorithms for analyzing the biomedical text that accompanies sequencing data. Its core utilizes the openly available AIONER platform, which is built upon the PubMedBERT model trained on the PubMed repository, to ensure efficient and accurate recognition of biomedical named entities (e. g., genes, diseases). This provides users with structured and meaningful keyword search results. By delivering a curated list of datasets, Alembic streamlines the path from search to analysis. Researchers can efficiently identify high-value targets and obtain a complete package of metadata and primary data to construct a tailored local repository. This positions Alembic as a universal solution that overcomes the fragmented approach of existing tools, offering an integrated workflow for diverse public sequencing data.

## Abstract (ru)

Развитие технологий высокопроизводительного секвенирования и методов анализа больших данных создает устойчивую потребность в повторном анализе накопленной в открытых репозиториях гетерогенной информации. Серьезной проблемой при этом остается преобладание свободного текстового описания биологических экспериментов, что затрудняет продуктивный поиск, систематизацию и дальнейшее использование соответствующих наборов данных. Прогресс в области искусственного интеллекта, особенно в развитии методов обработки естественного языка (natural language processing, NLP), обуславливает новые методологические возможности для эффективного решения этой задачи. Интегрированная система баз данных Entrez, поддерживаемая Национальным центром биотехнологической информации США (NCBI), предоставляет развитый и надежный доступ как к исходным данным секвенирования, так и к сопутствующей метаинформации, включающей детальное описание параметров экспериментов, через программный интерфейс (application programming interface, API). Это позволяет идентифицировать и загружать данные секвенирования и соответствующие им метаданные с описаниями экспериментов, используя поиск по ключевым словам и различным терминам, таким, например, как имена генов, в репозиториях; преобразовывать и систематизировать текстовые описания с применением современных NLP-методов и обеспечивать исследователям структурированную информацию для интеграции в локальные базы данных и форматированный перечень ссылок для загрузки исходных данных. Программный пакет Alembic предлагает комплексное решение для поиска и загрузки данных, автоматизируя все указанные этапы. Платформа использует клиент-серверную архитектуру и предназначенa для локальной установки. Для анализа биомедицинских текстов, сопровождающих данные секвенирования, в Alembic интегрированы современные алгоритмы искусственного интеллекта на основе архитектуры трансформеров. В частности, используется имеющаяся в открытом доступе платформа AIONER, обученная на данных репозитория PubMed с помощью модели PubMedBERT. Такой подход обеспечивает эффективное распознавание именованных сущностей (named entity recognition, NER) биомедицинского характера (гены, заболевания и др.), предоставляя пользователю структурированные результаты поиска по ключевым словам. Формируемый пакетом список дает возможность исследователю анализировать результаты, отбирать наиболее релевантные наборы данных и получать всю необходимую информацию (включая исходные данные) для создания локального репозитория, ориентированного на конкретную исследовательскую задачу. В отличие от имеющихся аналогов, Alembic является универсальным решением для интеграции данных из репозиториев открытого доступа и работы с разнородными типами данных секвенирования.

## Introduction

The rapid accumulation of sequencing data from multiple modalities, including WGSeq, RNASeq, and BSSeq, as well as related publications, is driven by advances in high-throughput sequencing technologies. The analysis of these extensive datasets calls for specialized software systems that can automatically and efficiently extract biologically relevant information. One promising approach involves the application of Natural Language Processing (NLP) algorithms. Modern NLP techniques automate the analysis of scientific texts, significantly enhancing the efficiency of large-scale data processing. A notable example is the MetaMap system (Aronson, Lang, 2010), which utilizes Named Entity Recognition (NER) to identify concepts from the Unified Medical Language System (UMLS) metathesaurus within textual sources. Building upon this foundation, specialized tools such as the BIONER package have further extended NER capabilities for biomedical entity recognition (Wang et al., 2019).

Building on the foundational role of NER in biomedical text extraction, advancements in neural network architectures and word embedding techniques have markedly improved the accuracy of biomedical entity recognition. The introduction of context-sensitive models, such as ELMo (Embeddings from Language Models), enabled the generation of word representations that incorporate contextual information. Recent progress in the field is characterized by the dominance of transformer architectures, particularly BERT (Devlin et al., 2019), which have transformed approaches to NLP tasks. Adapting these architectures for biomedical applications has spurred the development of domain-specific models, including BioBERT and PubMedBERT (Lee et al., 2020), as well as specialized processing frameworks such as scispaCy (Neumann et al., 2019).

The most extensive integrated system of biomedical databases remains Entrez, developed by the US National Center for Biotechnology Information (NCBI). This platform consolidates 38 distinct repositories, including PubMed, PMC, and databases of nucleotide and protein sequences. Data are added to the system through both automated processes, such as the analysis of scientific publications, and manual submissions by researchers. Depositing sequencing data into public archives (e. g., SRA, ENA) requires ones to provide essential metadata describing their experiments and biological samples. A principal advantage of Entrez is its programmatic interface, the Entrez Programming Utilities (E-utilities) (Sayers, 2022), which supports advanced querying and automated data extraction.

A common challenge in genomics involves identifying relevant raw datasets from public repositories. While programmatic access to resources like Entrez enables data retrieval, the integration of contemporary NLP for analyzing the associated biomedical text is key to building a unified system for this task. Existing tools, such as iSeq (Chao et al., 2024) and SampleExplorer (Chin, Lassman, 2024), have a narrow focus and offer limited functionality in this regard. To address this gap, we present Alembic, a system designed to analyze data within NCBI’s public repositories and perform the structured extraction of targeted information. Its name reflects its core function: much like the distillation apparatus used to extract essential substances from raw materials, Alembic refines meaningful insights from large volumes of primary data

## Materials and methods

**Fig. 1.**
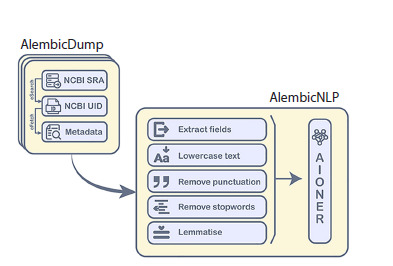
Schematic diagram of the Alembic package, comprising the AlembicDump (search result preprocessing) and AlembicNLP (named entity extraction) modules

Alembic employs a client-server architecture, where a client module handles query construction and result visualization, and a server module is responsible for processing the request and extracting the named entity. Figure 1 illustrates the overall system module design and data processing workflow.

The system operates on data from the NCBI Short Read Archive (SRA), accessed through the public Entrez API, allowing users to search using a broad spectrum of biomedical terms. Upon query execution, the AlembicDump module processes the results through a sequential pipeline: it first extracts NCBI Universal Identifiers (UIDs), uses them to fetch the full corresponding metadata in XML format, and then standardizes and restructures this information into a tabular format with predefined columns. This final table adheres to the Entrez SRA metadata schema, which comprises 21 fields containing either free-text descriptions (e. g., Study Abstract) or formally structured identifiers (e. g., Sample Accession).

The structured data from AlembicDump are then analyzed by the AlembicNLP algorithm to perform named entity recognition. First, the input text undergoes preprocessing with sciSpaCy (v 2.0.18), involving conversion to lowercase, removal of special characters and punctuation, filtering of isolated digits and stop-words (using the NLTK dictionary, www.ntlk.org, v 3.8.1), lemmatization, and tokenization. The processed text is then analyzed by the core AIONER model (Luo et al., 2023), a Bioformer-based architecture.

Alembic implements this using the embedded, opensource AIONER code with the bioformer-cased-v1.0 and BiomedNLP-PubMedBERT-base-uncased-abstract model variants, which were pre-trained on PubMed abstracts (https:// huggingface.co/lingbionlp/AIONER-0415/tree/main). These models were trained on information available in the article abstracts deposited in the PubMed catalog (Luo et al., 2023). The recognition stage outputs entities that are automatically categorized into predefined classes: Gene, Disease, Species, Cell Line, Variant, and Chemical. Consequently, AlembicNLP produces its final output: an annotated list that classifies each entity and records its textual coordinates.

The front-end application is implemented on the Vite JS framework (v 7.0) to optimize the build process. Its component- based architecture is developed in React (v 19), while the UI layer employs Material UI (v 7) components complemented by the MUI X DataGrid for advanced data table features. Asynchronous API communication is facilitated by the Axios library (v 1.6.7).

The application implements a clear workflow through two dedicated modules. The workflow begins with the Search Interface Module, where users query biomedical terms. This module returns results in a consolidated, paginated table optimized for large datasets.

The user then selects relevant fields, triggering the Data Preparation Module. This module automatically extracts and categorizes key terms for AI-driven analysis. This entire process is enabled by a client-server architecture: all complex data processing occurs on the server, while the client provides a clean, structured interface for interaction and presentation. This separation streamlines the analysis of complex descriptions and significantly reduces the user’s cognitive load.

The system presents an interactive dashboard for grouped results, equipped with hierarchical term filters, a faceted quick-search field, and dynamic highlight-on-hover functionality. These tools enable drill-down analysis and rapid identification of patterns within large datasets, eliminating the need to manually parse complete experimental descriptions. The interface implements a fully responsive design, ensuring optimal usability and consistent functionality across desktop, tablet, and mobile platforms.

**Fig. 2.**
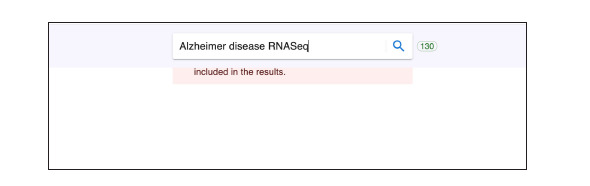
The Alembic search interface.

A researcher using Alembic typically follows a three-stage workflow: beginning with query formulation, followed by metadata structuring, and concluding with entity extraction. For instance, a search for Alzheimer’s disease transcriptomic data starts by entering keywords such as “Alzheimer”, “disease”, and “RNASeq” into the dedicated search interface (Fig. 2). The system returns the number of matching SRA records and, if results exceed 1,000 entries, prompts the user to refine terms or accept the default limit.

**Fig. 3.**
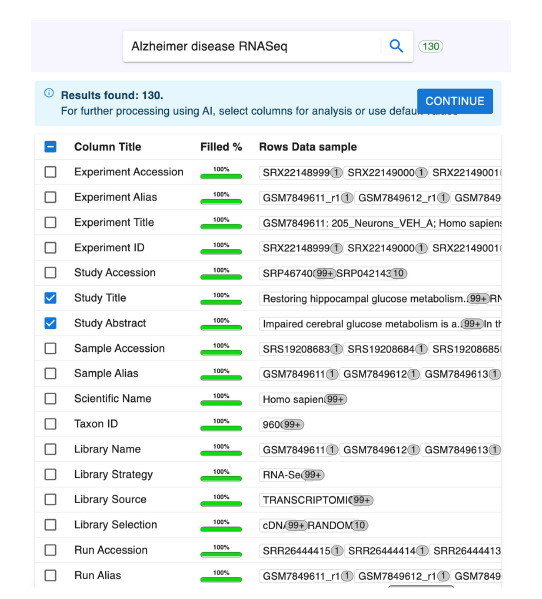
Aggregated, tabular view of the results returned from a keyword search.

In the second stage, Alembic structures the retrieved metadata into a table for review (Fig. 3). The table presents standard Entrez metadata field names, the percentage of experiments containing each field, and sample data entries. Using checkboxes in the first column, users can select multiple metadata types for subsequent AI-powered analysis.

**Fig. 4.**
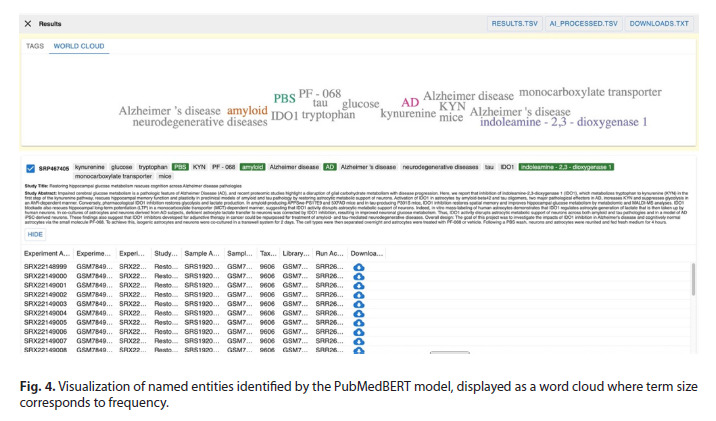
Visualization of named entities identified by the PubMedBERT model, displayed as a word cloud where term size corresponds to frequency.

Upon clicking the CONTINUE button, the system executes the NLP pipeline and displays the final output (Fig. 4). The results in this example are presented as a word cloud, visualizing the frequency of recognized entities. The interface includes download links in the upper-right corner for two tab-separated value (TSV) files: RESULTS.TSV (containing data prior to AI processing) and AI_PROCESSED.TSV (containing the postprocessed results). These structured files are suitable for direct import into standard relational databases, including SQLite, MySQL, or PostgreSQL. A separate file, DOWNLOADS.TXT, supplies direct URLs for retrieving the corresponding raw sequencing data from the SRA archive. The page concludes with a detailed listing of each experiment and its associated downloadable sequencing samples.

The project’s front end is built using modular JavaScript. Its architecture prioritizes scalability, allowing new functional modules to be added without modification of the core system. All source code and comprehensive documentation are publicly accessible under the MIT license via the project’s GitHub repository: https://github.com/shitohana/Alembic.

A local installation of Alembic requires Python v 3.12 or later and Docker v 24.0.2 or later. The setup procedure automatically installs all necessary dependencies, such as the TensorFlow (v 2.3.0), Transformers (v 4.18.0), and stanza (v 1.4.0) packages required for the integrated AIONER model. Once installation is complete, the system becomes accessible through a local web interface

## Results and discussion

The increasing scale of biomedical text processing has necessitated the development of numerous named entity recognition (NER) systems. Leading solutions in this area include scispaCy and AIONER. scispaCy provides models pretrained on biomedical corpora such as GENIA and MedMentions, enabling efficient handling of essential NLP tasks, including tokenisation, part-of-speech (POS) tagging, syntactic parsing, and NER, while accounting for domain-specific linguistic features. POS tagging, a foundational NLP technique, assigns grammatical categories (e. g., noun, verb, adjective) to each word in a text.

In contrast, AIONER specialises exclusively in biomedical NER (BioNER) through an innovative All-in-One (AIO) approach, which simultaneously recognises terms drawn from multiple annotated datasets. Unlike conventional methods that label tokens separately per category (e. g., Gene, Disease), the AIO architecture processes all categories concurrently using specially designed boundary tags. This reduces attribution errors for individual words or compound expressions, improves tokenisation accuracy, and addresses common challenges such as overfitting and poor generalisation, often stemming from sparsely annotated biomedical data. Built on modern pretrained language models like PubMedBERT and enhanced with a conditional random fields (CRF) layer for precise entity boundary detection, AIONER was selected as the core NER engine for the Alembic package due to these combined strengths.

The growing volume of biomedical text has driven the development of numerous named entity recognition (NER) systems, with scispaCy and AIONER being leading examples. scispaCy offers models pretrained on biomedical corpora, such as GENIA and MedMentions. These models efficiently perform essential natural language processing tasks, namely tokenisation, part-of-speech (POS) tagging, syntactic parsing, and NER, while incorporating domain-specific linguistic features. Here, POS tagging refers to the foundational NLP technique of assigning a grammatical category (e. g., noun, verb, adjective) to each word in a text.While tools like iSeq and SampleExplorer retrieve data from public repositories, Alembic introduces a fundamen- tally different approach grounded in natural language processing. The distinction reflects their core operational philosophies: iSeq functions as an automated data retrieval utility, whereas Alembic is designed for semantic analysis and entity extraction. iSeq operates via the command line to download batches of NGS data from major repositories, such as GSA, SRA, ENA, and DDBJ, using known accession numbers. Although it automates the download process and verifies data integrity, it performs no semantic analysis of metadata, relying entirely on pre-defined lists of identifiers. This approach treats data fundamentally as files to be transferred rather than information to be understood. SampleExplorer adopts a different strategy, employing language models to query repositories such as ARCHS4 for transcriptomic data. Its algorithm identifies semantically similar experiments by combining vectorised text metadata with transcriptomic similarity scores, using genes or textual descriptions as search queries. This design optimises discovery at the experiment level, enabling researchers to find analogous samples and related studies. However, it does not parse individual descriptions to extract and classify specific biomedical entities – such as genes, variants, and chemicals – which form the core function of Alembic’s NLP pipeline.

Unlike iSeq and SampleExplorer, Alembic provides a fundamentally distinct solution through an integrated NLP stack purpose-built for biomedicine. It utilises established preprocessing frameworks, notably scispaCy, and incorporates transformer architectures, including BERT and BioBERT. Its primary advantage, however, derives from implementing PubMedBERT (a model further trained on the entire PubMed/ PMC corpus) within the AIONER architecture (Luo et al., 2023). This specialised training enables Alembic to substantially outperform generic models, achieving superior accuracy in recognising complex biomedical terminology and interpreting its contextual meaning, thereby transforming unstructured text into structured, actionable knowledge.

## Conclusion

Alembic enables systematic extraction and structuring of biomedical metadata from open repositories through deep NLP-driven analysis. The system generates import-ready metadata packages compatible with SQLite and PostgreSQL, automates retrieval of linked raw sequencing files, and supports on-demand generation of specialized, project-specific local databases. The integrated AIONER platform leverages stateof- the-art language models, particularly PubMedBERT pretrained on the PubMed/PMC corpus, to accurately identify and classify biomedical entities with higher precision than alternative tools. Unlike command-line utilities that require manual query formulation, Alembic’s graphical interface and interactive visualizations streamline data discovery, reducing operational friction and enabling researchers to focus on analysis rather than data wrangling.

By converting unstructured metadata into structured, machine- actionable knowledge, Alembic substantially reduces the time and expertise required to prepare public repository data for downstream analysis.

## Conflict of interest

The authors declare no conflict of interest.
